# *Candida* Biofilms: Threats, Challenges, and Promising Strategies

**DOI:** 10.3389/fmed.2018.00028

**Published:** 2018-02-13

**Authors:** Mafalda Cavalheiro, Miguel Cacho Teixeira

**Affiliations:** ^1^Department of Bioengineering, Instituto Superior Técnico, Universidade de Lisboa, Lisbon, Portugal; ^2^iBB – Institute for Bioengineering and Biosciences, Biological Sciences Research Group, Instituto Superior Técnico, Universidade de Lisboa, Lisbon, Portugal

**Keywords:** *Candida* genus, biofilm formation, biofilm regulators, antibiofilm strategies, antifungal resistance, multi-species biofilm

## Abstract

*Candida* species are fungal pathogens known for their ability to cause superficial and systemic infections in the human host. These pathogens are able to persist inside the host due to the development of pathogenicity and multidrug resistance traits, often leading to the failure of therapeutic strategies. One specific feature of *Candida* species pathogenicity is their ability to form biofilms, which protects them from external factors such as host immune system defenses and antifungal drugs. This review focuses on the current threats and challenges when dealing with biofilms formed by *Candida albicans, Candida glabrata, Candida tropicalis*, and *Candida parapsilosis*, highlighting the differences between the four species. Biofilm characteristics depend on the ability of each species to produce extracellular polymeric substances (EPS) and display dimorphic growth, but also on the biofilm substratum, carbon source availability and other factors. Additionally, the transcriptional control over processes like adhesion, biofilm formation, filamentation, and EPS production displays great complexity and diversity within pathogenic yeasts of the *Candida* genus. These differences not only have implications in the persistence of colonization and infections but also on antifungal resistance typically found in *Candida* biofilm cells, potentiated by EPS, that functions as a barrier to drug diffusion, and by the overexpression of drug resistance transporters. The ability to interact with different species in *in vivo Candida* biofilms is also a key factor to consider when dealing with this problem. Despite many challenges, the most promising strategies that are currently available or under development to limit biofilm formation or to eradicate mature biofilms are discussed.

## Introduction: The Threats

A biofilm consists in a community of microorganisms that are irreversibly attached to a given surface, inert material, or living tissue, producing extracellular polymers that provide a structural matrix (1, 2). The microorganisms in this type of community exhibit lower growth rates and higher resistance to antimicrobial treatment, behaving very differently from planktonic cells (2). The ability to adhere to different types of surfaces enables microorganisms to form biofilm on medical devices, such as intravascular catheters, prosthetic heart valves and joint replacements, or in different tissues in the host (3), linking biofilms to persistent colonization and infections (4). A single microbial species is able to form biofilm although a mixture of bacterial and fungal species usually underlies biofilm formation *in vivo* (3, 5). Given that 80% of all microorganisms live in this form, biofilm formation becomes an irrevocable field to explore (6).

Due to these general characteristics, biofilms potentiate the establishment of unyielding infections in the human host. This is the case of biofilms formed by *Candida* species, causing superficial and systemic fungal infections in immunocompromised patients (7). These infections are very difficult to treat due to the characteristics of these species: resistance to antifungal drugs, expression of virulence factors, and the ability to form biofilm. Indeed, mucosal infections involve biofilm formation (8), usually including the interaction with commensal bacterial flora and host components (9). *Candida* species are the fourth most common cause of nosocomial bloodstream infection in the United States. These infections are associated with a high mortality rate of approximately 50% (10, 11). Biofilms are an additional problem since they are usually found in medical devices, such as prostheses, cardioverter defibrillators, urinary and vascular catheters, and cardiac devices (12, 13), hindering the eradication of *Candida* infections.

These challenging infections may be caused by different *Candida* species. *Candida albicans* is the most frequent pathogen responsible for *Candida* infections, followed by *Candida glabrata* (14). *Candida tropicalis* is particularly relevant in urinary tract infections (15) while *Candida parapsilosis* is frequently found in the skin of healthy hosts, being the causative agent of catheter-related infections (16, 17). Each *Candida* species exhibits differences in terms of biofilm formation, namely at the level of their morphology, characteristics of the extracellular matrix (ECM), and ability to confer antifungal resistance (18). This variability increases the challenge of finding an effective solution to tackle the threats of *Candida* biofilms as a unique problem. In fact, due to the emergence of these fungal infections, there is an urgent need to find adequate therapeutic approaches that might be able to treat patients more efficiently. The path to find these therapeutics certainly involves the study of the different pathogenic features of these species such as biofilm formation.

Biofilm formation, although being a process present in all the *Candida* species focused above, differs significantly from species to species, and in the dependency of surface, host niche and other factors. For example, *C. albicans* mature biofilms exhibit a more heterogenous structure, composed by blastophores and hyphae surrounded by an ECM of polysaccharide material (19). The ECM provides structural scaffold for adhesion between cells and with different surfaces, and a barrier between the cells in the biofilm and the neighboring environment (20). Within the structure of these biofilms there is usually water channels surrounding the microcolonies (21). In the case of *C. glabrata*, the biofilm is exclusively composed by yeast form cells in a multilayer structure intimately packed or in clusters of cells (22). In turn, *C. tropicalis* biofilm corresponds to a network of yeast, pseudohyphae, and hyphae, with intense hyphal budding (23), while *C. parapsilosis* exhibits a biofilm formed by clusters of yeast cells adhered to the surface, with minimal ECM (24). These differences highlight the complexity of the processes underlying biofilm formation and the difficulty to find a unique way to eradicate all *Candida* biofilms. *Candida* biofilms occur mostly in the mucosa or endothelium being involved in the development of common candidiasis, such as vaginal and oral candidiasis, but also associated with medical devices, such as vascular and urinary catheters and dentures (25). Interestingly, however, in all cases, biofilm formation compromises antifungal treatment and, when occurring in implanted medical devices, such as central venous catheters, implant replacement is often required (6, 26).

This review highlights the main challenges found in the process of developing effective therapy against *Candida* biofilms, considering differences found in biofilm formation and its regulation, as well as antifungal resistance found in biofilms and the establishment of mixed-species biofilm. Currently proposed promising strategies are also discussed herein.

## The Challenges

### The Complex Process of Biofilm Formation in *Candida* Species

Biofilm formation is a multifaceted process well described for *C. albicans*. In this case, the early phase of biofilm formation starts with adhesion of yeast cells to a given surface, followed by the formation of a discrete colony. Afterwards, in the intermediate phase, cells become organized and start producing and secreting extracellular polymeric substances (EPS). These components allow the maturation of a three-dimensional structure, forming the biofilm as we know it, in the maturation phase. Once the mature biofilm is formed, there is still the possibility of dissemination of progeny biofilm cells that become detached migrating to other niches to form more biofilm (18, 27).

For *C. albicans in vitro* biofilm formation, the early phase takes approximately 11 h, leading to the formation of distinct microcolonies. The intermediate phase of biofilm formation may last for 12–30 h, being characterized by the production of EPS and the formation of a bilayer usually composed by yeast, germ tubes and/or young hyphae. After this phase, *C. albicans* biofilm maturation includes the development of a thick layer of EPS where yeasts, and hyphae are present, forming a dense network. The maturation process usually takes approximately 38–72 h (18, 19, 27). Following maturation comes the dispersal phase, where mature biofilms release budding daughter cells as non-adherent yeast cells to propagate the colonization/infection (28).

It is important to highlight that most studies on biofilm formation have been conducted *in vitro*. A good example of an *in vivo* study was that conducted by Andes et al. that followed the development of *C. albicans* biofilm in a rat central venous catheter (29). This study reported important differences in biofilm formation between *in vivo* and *in vitro* experiments. For instance, the duration of the early phase of biofilm formation *in vivo* was found to be smaller than that observed *in vitro*, with several layers of yeast cells and hyphae being already present after 8 h of *in vivo* biofilm formation (29). Furthermore, maturation of the *C. albicans* biofilm *in vivo* was observed at 24 h in the rat central venous catheter and in an avascular implantation of small catheter in rats, contrary to the 38–72 h observed *in vitro* (29, 30). In the case of *C. glabrata, in vivo* maturation of the biofilm was only observed after 48 h in serum-coated triple-lumen catheters in a rat subcutaneous model. Although the early phase was similar between *in vivo* and *in vitro* conditions, after 24 h *in vitro* biofilms present a confluent layer of yeast cells while *in vivo* biofilms only exhibit patches of aggregated yeast cells. Moreover, *in vivo C. glabrata* biofilm development is dependent of the number of cells attached to the catheter, while *in vitro* it is not. The thickness of *C. glabrata* biofilm, 75–90 + 5 µm, is approximately half of the thickness of *C. albicans* biofilm (31).

The first crucial step of biofilm formation is adhesion. This process relies on several cell wall proteins, called adhesins, that promote the attachment to other cells, both to epithelial and to other microbial cells, or abiotic surfaces by binding to specific amino acid or sugar residues. Generally, adhesins are glycosyl-phosphatidylinositol-cell wall proteins (GPI-CWPs), comprising a GPI anchor, a serine/threonine domain and a carbohydrate or peptide binding domain (32). In *C. albicans*, several adhesins belong to the Als (agglutinin-like sequence) family, which is part of the GPI_CWP family. Such adhesins are known to bind to several proteins through its C-terminal region. Among the 8 members of the Als family, Als3 has the most prominent role in biofilm formation, as its deletion leads to severe biofilm defects when compared to the wild-type parental strain (33). *ALS*-like proteins have also been described to be present in other *Candida* species, such as *C. parapsilosis, C. tropicalis, C. dubliniensis, C. lusitaniae*, and *C. guilliermondii* (34), although still very little is known about the role of these adhesins in each species. Another important family of adhesins in *C. albicans* is the hyphal wall protein (Hwp) family, including Hwp1. Hwp1 is a mannoprotein of the cell wall of germ-tubes and hyphal cells, with a role in biofilm formation (35, 36). Besides Hwp1, also four other proteins in the Hwp family are required for biofilm development: Hwp2, Rbt1, Eap1, and Ywp1 (34). In *C. glabrata*, the EPA (epithelial adhesion) family of adhesins is the main responsible for adhesion. Most of these are encoded by genes localized in subtelomeric regions, including the *EPA1, EPA2, EPA3*, and *HYR1* cluster, and the *EPA4* and *EPA5* cluster (37). Although the EPA family in *C. glabrata* is predicted to comprise 17–23 genes, *EPA1, EPA6*, and *EPA7* genes are described as the most important in adhesion (35).

After adhesion, biofilm development continues through morphologic modifications, increase in cell number and production of EPS, influencing the final biofilm architecture. Analysis of the development of biofilm and production of ECM in *Candida albicans, C. parapsilosis, C. tropicalis*, and *C. glabrata* isolates of different origins have highlighted the differences in biofilm formation between species. Biofilms of *C. albicans* are more confluent than other *Candida* species biofilms (18), exhibiting different morphologic forms: oval budding, continuous septate hyphae and pseudohyphae, in infected tissues (3). On the surface of plastic coverslips, *C. albicans* biofilms exhibit a dense network of yeasts and filamentous cells embedded in a matrix of exopolymeric material (38). *C. albicans* is also considered to be the biggest biofilm producer among the *Candida* species focused herein (39). *C. glabrata* biofilms exhibit an ECM with high levels of carbohydrates and proteins (22), while isolates from the same species were observed to exhibit a scant population of blastospores, in silicone elastomer sheets (39). On the other hand, *C. parapsilosis* biofilm structure may vary according to the strain in study but usually comprises yeast and pseudohyphae morphologies, producing a compact multilayer or non-contiguous cell aggregates. The ECM of *C. parapsilosis* biofilms has been shown to be mainly composed by carbohydrates with low levels of proteins (22). Nevertheless, other evidences show that *C. parapsilosis* biofilms are the ones with less ECM produced when compared to *C. albicans, C. glabrata*, and *C. tropicalis* biofilms (39). Regarding *C. tropicalis*, a biofilm formation analysis revealed a structure composed mainly by yeast shaped cells, although some strains have exhibited filamentous forms in thick biofilms of coaggregated cells or in a discontinuous monolayer of yeasts anchored to the surface (22, 23). One specific isolate was shown to produce biofilm with a thin layer of matrix-encased hyphae (39). Although *C. tropicalis* biofilms have an ECM with a low content of carbohydrates and proteins (22), they are more resistant to detachment of the surface than those formed by *C. albicans* (40). When comparing biofilm production by *C. albicans, C. parapsilosis*, and *C. tropicalis* invasive isolates and *C. glabrata* blood isolates with non-invasive isolates, a significant higher production is observed in first isolates for all species, except for *C. tropicalis* (39).

Formed biofilms are also dependent on the EPS that are produced, which give a gel-like hydrated three-dimensional structure to the biofilm where the cells become partially immobilized (41). EPS plays different roles such as defense against phagocytosis, scaffold for biofilm integrity and prevention of drug diffusion. The production of EPS is dependent on the species and strain the carbon source and the rate of medium flow. For instance, it is known that *C. tropicalis* has a higher production of EPS matrix than *C. glabrata*. On the other hand, the increase of flow rate of the medium has shown to increase significantly the formation of the matrix (40). Usually, the major component of *Candida* biofilm EPS are polysaccharides (nearly 40%), although some differences are observed according to each *Candida* species (19, 40). In *Candida albicans* and *C. tropicalis*, the major carbohydrates present in the biofilm are glucose and hexosamine (39.6 and 27.4%, respectively). Proteins, phosphorus, and uronic acid may also be present but in small amounts (40). A biochemical analysis of *C. albicans* biofilm matrix in *in vitro* and *in vivo* models led to the identification of the following distribution of macromolecular components: 55% of proteins and their glycosylated counterparts, 25% of carbohydrates, 15% of lipids, and 5% of noncoding DNA. α-1,2-Branched α-1,6-mannans were the more abundant polysaccharides found associated with unbranched α-1,6-glucans, forming a mannan–glucan complex on the matrix (42). Although being relatively minor components of the ECM of *C. albicans*, β-1,3-glucan and β-1,6-glucan are also important components of this structure. Together with mannan, an interdependency between the synthesis or incorporation of these compounds seems to take place in the ECM since the inhibition of one of these three components leads to the decrease of the concentration of the others. Moreover, mannan and β-1,6-glucan, seem to be essential for ECM formation since the single deletion of genes encoding proteins involved in their production (*ALG11, MNN9, MNN11, VAN1, MNN4-4, PMR1*, and *VRG4* for mannan production and *BIG1* and *KRE5* for β-1,6-glucan production) nearly led to the complete elimination of the biofilm matrix (43). The majority of the identified lipids were neutral glycerolipids, polar glycerolipids and, in a smaller percentage, sphingolipids (42).

**Figure 1:**
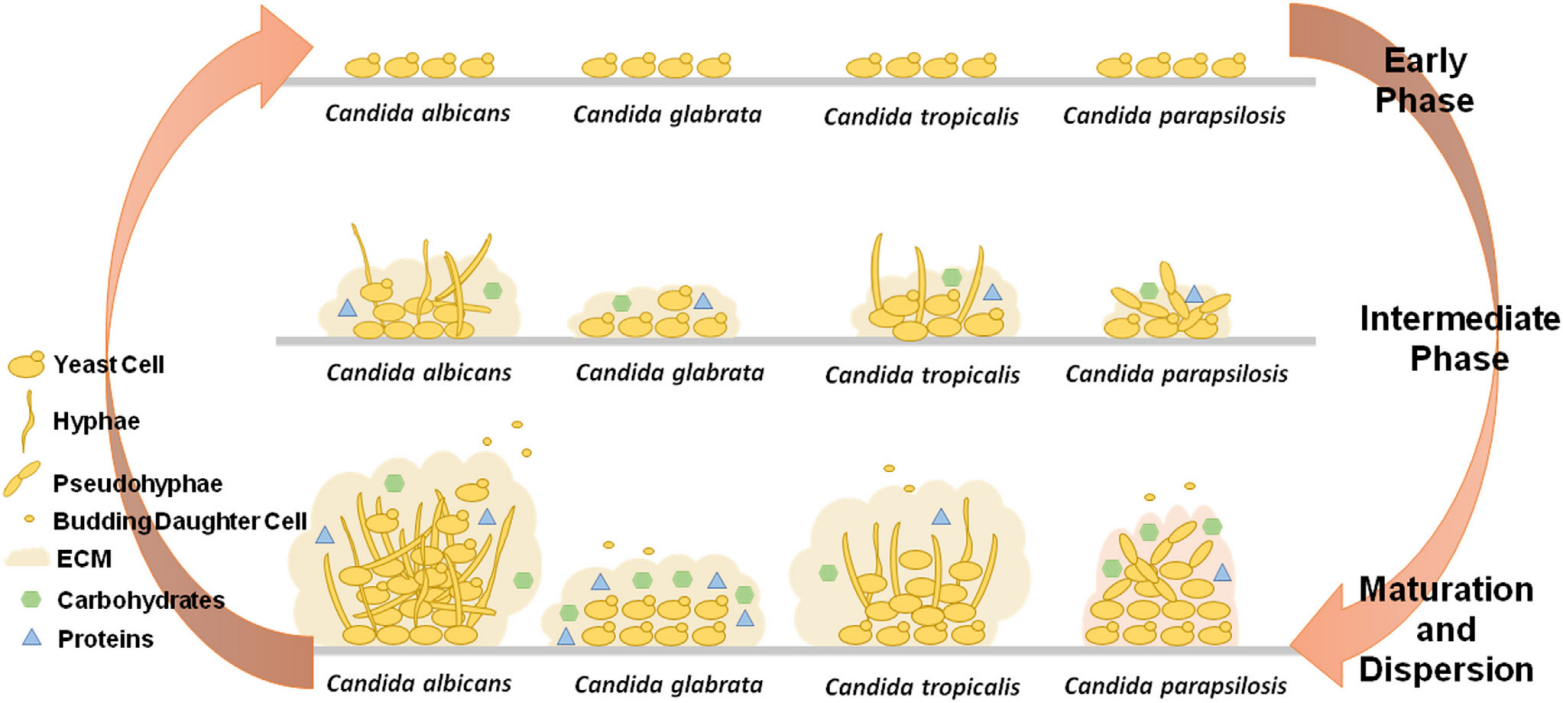
Comparative schematics the three stages of biofilm formation by *Candida albicans, Candida glabrata, Candida tropicalis*, and *Candida parapsilosis*, highlighting the different capacities to produce extracellular matrix (ECM), the varying components present in the ECM, and the ability to exhibit different cell morphologies.

*Candida* biofilms have thus a variety of possible architectures, adhesion properties, cellular morphologies and EPS composition, as illustrated in Figure 1, which are, not only species-, but in some cases strain-dependent, that turn the process of fighting these structures clinically very difficult. To increase the complexity of the problem, several other external factors related to the environment surrounding the biofilm also influence the final biofilm produced.

### External Factors Influencing Biofilm Development

Each step of biofilm formation depends on the environmental conditions experienced by *Candida* species (Table 1).

**Table 1:** Factors influencing *Candida* biofilms.

	Factors influencing *Candida* biofilms							
	EPS	Adhesins	Transc. factors	Preferred substratum	Preferred carbon source	Drug resistance		
						Drug	Genes involved	Model/evidence
*Candida albicans*	Polysaccharides glucose hexosamine lipids, proteins phosphorus uronic acid non-coding DNA	Als1-7 Als9, Hwp1, Hwp2, Rbt1, Eap1, Ywp1	Efg1, Bcr1, Tec1, Ndt80, Rob1, Brg1, Cph1, Nrg1, Tup1, Ume6, Cup9, Slf1, Rfg1, Csr1, Gcn4, Tye7, Rca1, Ace2	Latex, silicon elastomer, Teflon	Glucose	Fluconazole, voriconazole, ravuconazole, amphotericin B, nystatin, chlorhexidine, terbinafine	–	Bioprosthetic model
						Amphotericin B, nystatin, fluconazole, chlorhexidine	–	Silicone elastomer model
						Fluconazole	*CDR1, CDR2, MDR1*	Plastic coverlips *in vitro*
						Fluconazole	*MNN9 MNN11, VAN1 MNN4-4, VRG1 PMR, BIG1 KRE5 FKS1*	*In vitro*
*Candida glabrata*	–	Epa1-7 Hyr1	Cst6, Ace2	Polyvinyl chloride	Glucose	Fluconazole	–	*In vitro* in over denture acrylic strips
						Caspofungin, amphotericin B, nystatin, ketoconazole, 5-flucytosine	–	*In vitro* over poly-styrene surface
*Candida tropicalis*	Polysaccharides glucose hexosamine proteins phosphorus uronic acid	Als-like proteins	Nrg1 Cup9 Slf1 Ume6 Tec1	Teflon, polyurethane	–	Fluconazole, amphotericin B	–	*In vitro* over poly-styrene surface
*Candida parapsilosis*	–	Cpar2_404800Als-like proteins	Ume6 Efg1 Bcr1 Cph2 Ace2	Teflon	–	Fluconazole, voriconazole, ravuconazole, amphotericin B, nystatin, chlorhexidine, terbinafine	–	Bioprosthetic model

*Main EPS produced, important adhesins, TFs regulating biofilm, most advantageous substratum and carbon source for biofilm formation and biofilm drug resistance found for Candida albicans, Candida glabrata, Candida tropicalis, and Candida parapsilosis*.

One important factor is the substratum to which the biofilm is attached. There are several options of substratum used to study *Candida* biofilms *in vitro*: polystyrene (44), polymethylmethacrylate (19), polyvinyl chloride (45), cellulose cylindrical filters (45), acrylic (46), and denture base materials (47) have been described for *C. albicans* biofilm formation, while silicone elastomer catheter disks have been tested for different *Candida* species (19, 48). Among these, hydrophobic surfaces lead to the formation of two phases in *C. albicans* biofilm. The first phase corresponds to the adhesion of blastospores to the surface and a second phase where hyphae elements are embedded in EPS with water channels that allow the diffusion of nutrients and passage of waste disposal between the bottom layer and the surface (19). On the other hand, substratum may influence the adhesion process itself, e.g., soft lining materials of dentures lead to higher adhesion when compared to acrylic surfaces (49). It also influences the size of the developed *Candida* biofilm (47). *C. albicans* biofilms are larger in latex and silicone elastomer surfaces, and smaller in polyurethane and on silicone, when compared to polyvinyl chloride (48). Chandra et al. have tested the effect of different modifications on the surface of biomaterials on the formation of *C. albicans* biofilms. This work has led to the discovery that polyetherurethane with 6% of polyethylene oxide significantly reduces the total biofilm biomass, as well as the metabolic activity of the cells in the biofilm (50). In turn, Estivill et al. have studied the biofilm formation of *C. albicans, C. glabrata, C. parapsilosis, C. tropicalis*, and *C. krusei* on polyvinyl chloride, polyurethane, and Teflon. All species exhibited increased biofilm formation on Teflon, except for *C. glabrata*, for which the best surface for biofilm formation was found to be polyvinyl chloride. *C. tropicalis* showed the highest number of cells recovered from biofilm development in a polyurethane catheter, while on polyvinyl chloride catheter, *C. parapsilosis* was the one with the highest number of cells (51). These differences in preference for surface material, that again appear to vary in a species- and strain-dependent manner, highlight the importance of selecting the most adequate biomaterials to prevent or, at least limit, the propensity for the development of biofilm-seeded infections by *Candida* species.

Besides the substratum, a relevant factor influencing biofilm formation is the available carbon source. When comparing the number of cells per biofilm formed by *C. albicans* and *C. glabrata*, a clear increase is observed when glucose is used as the carbon source, comparatively to sucrose (52) or, in the case of *C. albicans*, also to galactose (44). The test of different concentrations of glucose, has revealed that even low concentrations of glucose induce *C. glabrata* biofilm (53). Moreover, the medium used to evaluate biofilm formation *in vitro* has also shown to be responsible for differences in morphology. On a polymethylmethacrylate surface, *C. albicans* biofilm cells have been shown to predominantly include yeast shaped cells in YNB medium, and hyphal cells in RPMI 1640 medium (19). This indicates that biofilm formation does not require a given morphology, reinforcing the fact that biofilm development is intrinsically dependent on the environmental conditions. The presence of saliva has been described as another influencing factor of biofilm formation in *Candida* species. Its addition to the medium leads to a significant decrease of *C. albicans* or *C. glabrata* biofilm formation (52). Nonetheless, in the presence of saliva *C. albicans* exhibits more biofilm formation than *C. glabrata, C. tropicalis*, and *C. parapsilosis*, while the later exhibits higher adhesion capacity to abiotic surfaces. On buccal epithelial cells, *C. glabrata* exhibits the highest capacity of adhesion (54).

Fluid flow rate also affects biofilm formation, affecting EPS production, among other factors. Hawser et al. observed that under static conditions, EPS production was minimal when compared to the use of liquid flow. In fact, increasing the speed of flow, the ECM produced was significantly higher, totally wrapping the cells of *C. albicans* biofilm, whose metabolic activity did not change with such alterations. Nevertheless, a shaking speed of 60 rpm was found to inhibit biofilm formation (55). Moreover, Al-Fattani and Douglas showed that *C. albicans* and *C. tropicalis* biofilms had more ECM produced when grown under conditions of continuous flow in a modified Robbins device than in static conditions (40). Furthermore, *C. albicans* biofilms under shear stress were found to become thinner but denser, when compared to static conditions, and once again metabolic activity did not suffer any modification (56).

### Regulation of Biofilm Formation in *Candida* Species

**Figure 2:**
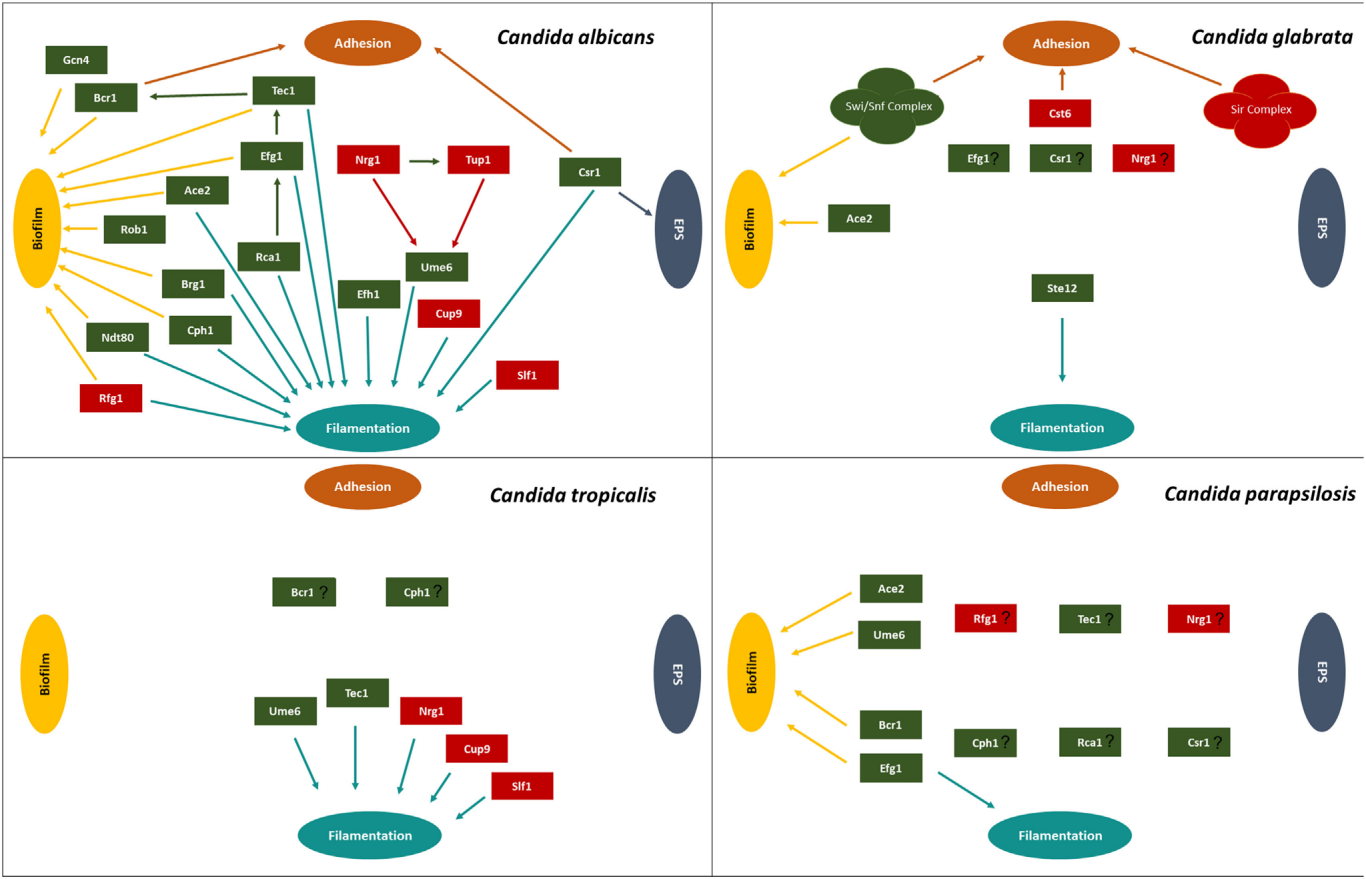
Transcription regulatory network described for *Candida albicans, Candida glabrata, Candida tropicalis*, and *Candida parapsilosis*, highlighting the transcriptional factors involved in adhesion, extracellular polymeric substances (EPS), filamentation, and biofilm. Green boxes indicate activators and red boxes indicate repressors. Participation of each transcription factor in these processes is indicated by the colored arrows: brown arrows correspond to adhesion, dark blue arrows correspond to EPS, light blue arrows correspond to filamentation, and yellow arrows correspond to biofilm formation.

Biofilm formation is regulated by several transcription factors, including those involved in adhesion, hyphal formation, and EPS production, as assembled in Figure 2 and Table 1. The study of the elaborated regulatory network that underlies biofilm formation is a promising field of research, as it is expected to lead to the discovery of the best targets to tackle biofilm formation by *Candida* species in the host.

Nobile et al. have identified important transcription factors essential for biofilm formation using a *C. albicans* library of 165 transcription factor deletion mutants that were tested for biofilm growth *in vitro* and *in vivo* on rat denture and catheter models (57). The biofilm defective deletion mutants identified were *efg1Δ/Δ, bcr1Δ/Δ, tec1Δ/Δ, ndt80Δ/Δ, rob1Δ/Δ*, and *brg1Δ/Δ*. Resorting to Chromatin ImmunoPrecipitation-on-chip, it was possible to unravel the promoter regions bound by each transcription factor during biofilm formation. The six transcription factors were found to regulate a high degree of overlapping target genes as well as to control the expression of each other (57).

In *C. albicans*, the main regulators of biofilm development are also involved in filamentous growth, given the typical hyphal differentiation that takes place during this process. That is the case of Efg1 and Cph1 that positively control the expression of genes required for hyphal growth, such as *ECE1, HYR1, HWP1*, and *ALS3* (58–62). These transcription factors also control the expression of cell wall-related genes due to the usual morphological alterations involved in the transition from yeast to hyphae (63, 64). The *Δefg1* and *Δefg1Δcph1* deletion mutants have been shown to be incapable of filamentation or biofilm development, being only able to form a sparse monolayer of adherent elongated cells (65). *C. albicans* Efh1 was found to be a homolog of Efg1, regulating, together with Efg1, a set of genes involved in filamentation (66). In *C. parapsilosis*, Efg1 has been shown to be a morphological switch regulator (67), and to have a role in biofilm formation since its absence leads to reduced biofilm (68). Surprisingly, in *C. glabrata*, which is unable to form hyphae, a homolog of Efg1, encoded by ORF *CAGL0L01771g*, has been found but remains uncharacterized (69). In turn, Cph1 is the terminal transcription factor of the MAP kinase pathway in *C. albicans*, mediating a feedback regulation of this pathway (62). This protein belongs to the STE-like transcription factor family being present in *C. albicans, C. tropicalis*, and *C. parapsilosis*. In *C. glabrata*, the Cph1 ortholog is the Ste12 transcription factor involved in the regulation of filamentation induced by nitrogen starvation (69, 70).

Also involved in filamentous growth regulation in *C. albicans* is the Ndt80 transcription factor. This regulator has also been associated with the control of cell separation, azole resistance, virulence and biofilm formation (57, 71, 72). Likewise, Brg1 has a role in the transcriptional control of hyphal growth-related genes in *C. albicans*, since the absence of both copies of the *BRG1* gene leads to impaired hyphal development. Meanwhile, its overexpression was found to lead to the increased expression of hyphae-specific genes, including *ALS3, HWP1*, and *ECE1*. *C. albicans* Brg1 is, in turn, repressed by the Nrg1 C2H^2^ zinc finger transcription factor, involved in the control of cell morphology and pathogenicity, by repressing filamentous growth (73, 74). The *C. tropicalis* Nrg1 homolog was similarly found to have a role in repressing filamentation (75), while other predicted homologs in *C. parapsilosis, C. tropicalis* and *C. glabrata* remain uncharacterized (69). Besides Nrg1, *C. albicans* and *C. tropicalis* count with Cup9 and Slf1 regulators to suppress filamentous growth (75). In turn, Ume6 transcription factor has also been found in *C. parapsilosis*, playing an important role in biofilm development (68), and in *C. albicans* and *C. tropicalis*, as a regulator of filamentous growth (75). Another negative regulator of filamentous growth and biofilm formation in *C. albicans* is Rfg1 (74, 76, 77). Together with Nrg1, Rfg1 down-regulates the expression of *ALS3, ECE1*, and *HWP1* hyphae-specific genes (78, 79).

Bcr1, a C_2_H_2_ zinc finger transcription factor, is also an important regulator of biofilm formation, but not of hyphal formation. Bcr1 regulates the expression of cell surface proteins encoded by the *HYR1, ECE1, RBT5, ECM331, HWP1, ALS3, ALS1*, and *ALS9* genes (80), controlling the adhesion process in *C. albicans*, during the early phase of biofilm formation. Moreover, the absence of Bcr1 in an *in vivo* rat model of catheter-based infection was found to abrogate biofilm formation after 48 h of infection. Interestingly, the overexpression of *ALS3* in a *bcr1/bcr1* background completely restored the biofilm formation phenotype (33). In *C. parapsilosis*, a homolog of Bcr1 was identified as one of the most important transcription factors regulating biofilm formation (68, 69, 81, 82). Bcr1 is itself positively regulated by the transcription factor Tec1, in *C. albicans* (80). Tec1 positively regulates morphogenesis and is essential for hyphae formation upon serum induction, macrophage evasion and expression of aspartyl proteinase genes (65, 83). In turn, Efg1 and Cph2 are known to regulate Tec1 (84, 85). *C. parapsilosis* has been found to have a homolog of Tec1 (69). Tec1 of *C. tropicalis* is an important transcription regulator promoting filamentation (75).

Regulation of biofilm matrix biogenesis has also been studied, although not so extensively. *C. albicans* Csr1 regulator is involved in the control of adherence (86) and development of the ECM of *C. albicans* biofilms (87). More specifically, Csr1 negatively controls the concentration of β-1,3-glucan present in the matrix by activating *CSH1* and *IFD6*, and inhibiting the expression of *GCA1, GCA2*, and *ADH5*. Csr1 has also been found to participate in the control of filamentous growth (87). On the other hand, a transcription factor that positively controls glycolytic genes, Tye7, was found to be essential for biofilm cohesiveness in *C. albicans* (88).

The Rca1/Cst6 in *C. albicans* is a regulator of hyphal formation by positively controlling the expression of hyphal genes like *GWP1, ECE1, HGC1*, and *ALS3* and the transcription factor Efg1 (89). In *C. glabrata*, Cst6 is another important transcription regulator of biofilm formation. This protein is a bZIP transcription factor of the ATF/CREB family that negatively controls the expression of the *EPA6* gene, which encodes an important adhesin found in *C. glabrata* biofilms (90).

In *C. albicans*, an Ace2 transcriptional regulator was also found conserving the C2H^2^ zinc finger domain (69). The absence of Ace2 leads to hyperfilamentation and hypervirulence in *C. albicans* (91, 92), although it has been shown to be required for biofilm formation in normoxia conditions (93) and filamentous growth under hypoxic conditions (94). The *C. parapsilosis* and *C. glabrata* Ace2 homologs also play important roles in biofilm formation (68, 95).

Although having specific transcription factors regulating biofilm formation, *C. glabrata* has a number of related genes whose expression is controlled by subtelomeric silencing. *HYR1, EPA1, EPA2, EPA3, EPA4, EPA5, EPA6*, and *EPA7* genes of *C. glabrata* have been found to be repressed by this process, given their loci proximity to a telomere. This regulation is mediated by the Sir complex (Sir2–Sir4), Rap1, Rif1, yKu70, and yKu80 (37, 96), as well as the Swi/Snf complex (90). Both Swi/Snf and Sir complexes appear to underlie the regulation of biofilm formation which is specific for *C. glabrata*, when compared with other *Candida* species.

Given the multifactorial nature of biofilm formation, its regulatory network is highly complex, involving the control of adhesion, hyphal formation, ECM production, and other processes (Figure 2). Finding a general regulator to tackle the regulatory control of biofilm in all *Candida* species is thus a very challenging goal.

### Antifungal Resistance through Biofilm Formation

Biofilm formation has been tightly linked to the development of resistance to antifungal drugs (Table 1). For example, resistance acquisition during biofilm formation was observed in a study where 48 h biofilms of *C. albicans* were found to exhibit five- to eightfold higher resistance to all antifungals when compared to planktonic cells (97). Increased resistance exhibited by *C. parapsilosis* biofilms to fluconazole, voriconazole, ravuconazole, amphotericin B, nystatin, chlorhexidine, and terbinafine have also been registered (98).

This enhancement of resistance when *Candida* species grow as biofilms might be explained by several factors. One of them is the increased metabolic activity occurring in the early development of biofilm. Chandra et al. have shown that although MICs of amphotericin B, fluconazole, nystatin, and chlorhexidine were 0.5, 1, 8, and 16 µg/mL, respectively, in the early stage of *C. albicans* biofilm formation on polymethylmethacrylate strips, after 72 h of biofilm formation, MICs of amphotericin B, fluconazole, nystatin, and chlorhexidine had changed to 8, 128, 32, and 256 µg/mL, respectively (19). Interestingly, however, the defective biofilm formation mutants, *Δefg1* and *Δefg1Δcph1*, which only exhibit an adherent monolayer of elongated cells, show the same resistance toward fluconazole and amphotericin B as the wild-type. These results suggest that resistance to antifungal drugs is related to adherence, regardless of normal biofilm formation (65).

Another issue to consider is the possible role of the ECM in antifungal drug resistance. Some authors advocate that EPS contributes as a barrier to prevent the diffusion of drugs, showing that *Candida* cells resist 20% better to amphotericin B with EPS, when compared to the same cells upon EPS removal (18). Indeed, the extracellular β-1,3-glucan matrix has been proven to bind to amphotericin B, while its absence from the matrix, increases *C. albicans* susceptibility to fluconazole and amphotericin B (99). Other evidences were given in a study where radiolabeled drug (^3^H-fluconazole) is only sequestrated in the presence of matrix mannan and β-1,6-glucan, demonstrating the importance of these molecules in antifungal resistance (43).

Alterations in gene expression during *C. albicans* biofilm formation include the upregulation of *CDR* and *MDR* genes encoding azole resistance transporters (38). This upregulation seems to be important for the development of antifungal resistance in the early phase of the biofilm formation, while in the maturation process, changes in sterol composition appear to be more relevant (18). Nevertheless, it has been shown that a set of isogenic *C. albicans* strains carrying single or double deletions of genes encoding efflux pumps (*Δcdr1, Δcdr2, Δmdr1, Δcdr1/cdr2*, and *Δmdr1/cdr1*) could form biofilm like the parental strains, still displaying antifungal resistance. This gives evidence that antifungal resistance in biofilm does not depend on a single mechanism (38). Interestingly, overexpression of *CDR1, CDR2* and *TPO1_2* genes has also been observed in *C. glabrata* biofilms (100, 101). Tpo1_2 is a major facilitator superfamily transporter known to have a role in antifungal resistance (102) and to be necessary for the normal expression of *ALS1, EAP1*, and *EPA1* genes involved in adhesion (101). In fact, *C. glabrata* biofilms have shown to be significantly more resistant than planktonic cells (100, 103).

Showing that some specific mechanism of biofilm formation might be behind the resistance to antifungal drugs, *ALS3* and *HWP1* were found to be induced upon exposure to caspofungin in *C. albicans* biofilms but not in planktonic growing cells (104). Additionally, the deletion of *MNN9, MNN11, VAN1, MNN4-4, VRG4, PMR1, BIG1, KRE5*, and *FKS1* genes was found to lead to higher susceptibility to fluconazole in biofilm cells, but not in planktonic cells (43). Interestingly, exposure to 80 µg/mL of fluconazole was found to lead to almost no changes in gene expression in SC5314 *C. albicans* biofilms, while exposure to caspofungin or amphotericin B results in massive gene expression changes, suggesting that biofilm cells were innately protected against azole drugs (104).

Similar to what has been described for other microorganisms, persister cells are also found within biofilms formed by *C. albicans*. Persister cells are dormant, non-dividing cells that have high tolerance to antimicrobial drugs. This tolerance is believed to be possible thanks to the dormancy of the cell, which enables the binding of antimicrobial drugs to their specific target, while making it impossible to the drug to inhibit the function of the target molecule. This tolerance, however comes at the price of non-proliferation (105). In *C. albicans*, biofilm persister cells display resistance to fluconazole, while the same cells grown in non-biofilm planktonic culture are sensitive to fluconazole. Interestingly, these persister cells cultivated in biofilm exhibit increased expression of the *CDR* genes (38). This phenomenon was also observed for *C. albicans* cells in biofilm exposed to amphotericin B and chlorhexidine, but independently of the expression of drug efflux pumps (106).

### Multi-species Biofilm Formation and Quorum Sensing

*Candida* species biofilms have been usually studied as single species biofilm, although *in vivo* a very different reality is observed. Generally, biofilms are composed by multiple species that interact as a community having synergistic and/or antagonistic relationships. The synergistic interactions involve metabolic cooperation while antagonistic interactions include competition for nutrients and the generation of inhibitory compounds (107).

The formation of mixed biofilms of *Candida* species has been described with different combinations between *Candida* species or with *Candida* and bacteria. *C. albicans, C. glabrata, C. krusei*, and *C. tropicalis* have been shown to form biofilm with each other (52, 108). Although increased biofilm formation was observed for most combinations of *Candida* species, for *C. tropicalis* this increase was only observed when in interaction with *C. albicans* (108).

*Candida albicans* biofilms have also been shown to be favored by combination with four species of bacteria: *Streptococcus mutans, Streptococcus sanguinis, Actinomyces viscosus*, and *Actinomyces odontolyticus*. Increased hyphae development, accompanied by the overexpression of *HWP1, ALS3*, and *EPA1*, was observed in mixed-species biofilm, both on acrylic surfaces or in reconstituted human oral epithelia (RHOE). Moreover, tissue damage and invasiveness is higher when all these five species are infecting and forming biofilm on RHOE. When compared to single *Candida* or single bacteria biofilms, mixed-species biofilm are significantly more invasive (109).

Multispecies biofilm formed upon denture stomatitis that frequently involves *C. albicans, S. mutans, S. sanguinis*, and *Actinomyces naeslundii* has also been investigated. Evidence of the increased pathogenicity of *C. albicans* in the presence of these bacteria was obtained, suggesting that in these conditions the use of antibacterial agents could have an effect in decreasing fungal proliferation (110). Interestingly, *S. mutants* and *C. albicans* have been shown to have a synergetic relationship since their coexistence leads to increased biofilm formation, when compared to single biofilms, despite the negative effect *S. mutants* has on hyphae formation. This dual-species biofilm displays two layers: *S. mutans* cells attached to the surface followed by *C. albicans* cells as a second layer (52). Interestingly, *S. mutans* was also reported to increase biofilm formation of *C. glabrata* (52). Multispecies biofilm formation on infected mice has also been investigated, considering *C. albicans* in coexistence with epithelial cells, neutrophils and commensal oral bacteria, such as *Enterococcus/Lactobacillus* sp. and *Staphylococcus* sp. (9).

*Staphylococcus aureus* interaction with *C. albicans* has been described in more detailed. Both species are able to cooperate in order to form a polymicrobial biofilm, even in the presence of serum which does not allow the formation of *S. aureus* single species biofilm. Visualization of these dual-species biofilm showed a first layer of *C. albicans* biofilm attached to the surface covered by a *S. aureus* monolayer that is included in the ECM formed by *Candida* cells. This ECM has proven to protect *S. aureus* from killing by vancomycin (111). This positive interaction has shown to be also dependent on hyphae formation by *C. albicans* (112). *Staphylococcus epidermis* has also been shown to cooperate with *C. albicans*, forming a multispecies biofilm that allows the protection of *S. epidermidis* against vancomycin and of *C. albicans* against fluconazole (40, 113) and amphotericin B (40).

When a synergistic relationship takes place, quorum sensing is an essential process that allows communication between species. Quorum sensing has been described mostly in bacteria, although several studies have already reported the use of quorum sensing between bacteria and *Candida* species (9, 114). This process comprises the production and release of a signal molecule (autoinducer), which, according to the cell density, will increase in concentration and orchestrate a collective and coordinated expression of given genes, in all the species involved for the development of biofilm (107). One example of quorum sensing has been described for the interaction between *C. albicans* and *Streptococcus gordonii*. These two species have been shown to coaggregate thanks to two cell wall anchored proteins of *S. gordonii*, SspA and SspB (114, 115), the later interacting directly with exposed *C. albicans* Als3 (116). The positive interaction between these two species is based on the production and release of a chemical molecule denominated autoinducer 2 by *S. gordonii* (117). This interaction has several advantages to *C. albicans*, such as the enhancement of hyphae formation and resistance to farnesol (114).

Farnesol is also a quorum-sensing molecule but produced as a secondary product of sterol biosynthesis and secreted by *C. albicans* (118). When accumulated, it prevents the formation of filamentous growth and biofilm (119–122). Generally, farnesol is mostly secreted in the later stages of biofilm formation by *C. albicans* (122). Among pathogenic *Candida* species, only *C. albicans* and *C. dubliniensis* are known to secrete farnesol (123). Farnesol is considered to be an autoinducer molecule that influences the expression of genes involved in antifungal resistance, cell wall maintenance, phagocytic response, surface hydrophobicity, iron metabolism and heat shock (120). Given its effect and synergistic action with antifungal therapy, it has been considered as a potential therapeutic agent (124). In *C. parapsilosis*, farnesol has also been described as having an inhibitory effect on growth and biofilm formation (125, 126). The presence of exogenous farnesol has been shown to lead to increased expression of oxidoreductases and a change in the expression of genes related to sterol metabolism (125). Moreover, reference strains of *C. parapsilosis, C. tropicalis, C. dubliniensis*, and *C. albicans* were found to have reduced biofilm production upon exposure to farnesol. However, *C. glabrata* and *Candida krusei* biofilm development was found to remain unaltered upon exposure of this quorum-sensing molecule.

Along with farnesol, a few other molecules produced by *C. albicans* have been proposed to work as quorum-sensing molecules, including morphogenic autoregulatory substance, phenylethyl alcohol and tryptophol (127), tyrosol, and farnesoic acid. Although through different pathways, farnesoic acid, just like farnesol, is involved in regulating the yeast-to-hyphae transition (119, 127). Depending on the strain, *C. albicans* might produce farnesol or farnesoic acid, both having an inhibitory effect of filamentous growth (127). Tyrosol, on the other hand, has an opposite effect on yeast-to-hyphae transition, increasing cell density and promoting the formation of germ tubes and the transition from yeast to hyphae (128). Tyrosol is produced by planktonic and biofilm cells, having a stimulating effect in hyphae formation in the early stage of biofilm development (122).

Interaction of *C. albicans* with *Aspergillus nidulans* has been shown to be competitive thanks to the production of farnesol. In fact, the presence of exogenous farnesol or the co-culture of *A. nidulans* with *C. albicans* activates apoptosis in *A. nidulans* cells. This activation takes place in a mitochondria dependent way, through the FadA G protein complex signal transduction pathway (129).

Another antagonistic relationship is the one described between *C. albicans* and *Pseudomonas aeruginosa*. In this specific case, *P. aeruginosa* is known to attach to *C. albicans* filaments, forming biofilm over the hyphae instead of the surface. The formation of biofilm ultimately leads to the death of *C. albicans* filamentous cells, although yeast cells remain viable (130). In fact, the presence of secreted factors by *P. aeruginosa* causes downregulation of genes involved in adhesion and biofilm formation, and increases the expression of genes encoding drug exporters, such as *CDR1* and *SNQ2*, and the *YWP1* gene encoding a protein involved in biofilm dispersal, in *C. albicans* biofilms (131). This negative effect over *C. albicans* is related to a quorum sensing molecule produced and secreted by *P. aeruginosa*, the 3-oxo-C12 homoserine lactone. Other compounds with a 12-carbon backbone, like dodecanol, have a similar effect on *C. albicans* filamentous growth (132). On the other hand, farnesol produced by *C. albicans* leads to a decreased quinolone signal from *Pseudomonas* that regulates the production of extracellular proteases, hydrogen cyanide and redox-active phenazines (133). According to Holcombe et al., the factors secreted by *P. aeruginosa* seem to have a specific effect in the maturation phase of biofilm formation by *Candida* species (131). *C. glabrata, C. tropicalis, C. parapsilosis*, and *C. dubliniensis* biofilm formation is also inhibited upon interaction with *P. aeruginosa* (134), which has a growth inhibitory effect on these *Candida* species (135, 136). Interestingly, *P. aeruginosa* proliferation is also inhibited in the presence of *C. albicans, C. glabrata*, and *C. krusei* (134).

## Promising Strategies

Although no definitive solution for *Candida* biofilms has been found yet, there are several promising strategies being implemented nowadays, as well as intense research being developed in the search for novel solutions that alone or together with others might become the answer to this problem.

The current therapies with partial success to fight this challenge are based on two different approaches. The first, called “lock” therapy is directed to eradicate biofilm formation in catheters prior to their contact with the patient. The strategy consists in diffusing a high concentration of an antimicrobial drug into the catheter lumen, letting the agent act during a period of several hours or days. This technique avoids systemic toxicity since the antimicrobial drug only acts in the catheter (137). Examples of success using the “lock” therapy are the tests performed on silicone catheters infected with different *C. albicans* and *C. glabrata* isolates, where micafungin (5 and 15 mg/L), caspofungin (5 and 25 mg/L), and posaconazole (10 mg/L) were used as antimicrobial agents. All antifungals used with this technique help decrease *Candida* biofilms, being micafungin the most effective (138). Nevertheless, when using amphotericin B lipid formulation (1,000 mg/L), known to have activity against *Candida* biofilms (98), in a “lock” therapy approach, an effective eradication of *Candida* biofilms was not observed (139). Moreover, an interesting antimicrobial agent tested in “lock” therapy is ethanol. Combined “lock” therapy using 0.3 mL of 70% ethanol and 5 mg/L of micafungin was effective eliminating a catheter-related candidemia in a male infant (140). Ethanol is an advantageous antimicrobial agent given its low cost and effectiveness against *Candida* species.

The other approach is catheter coating. Modification of the coating of the catheter with minocycline-rifampin, chlorhexidine, or silver sulfadiazine decreases the number of bloodstream infections caused by central venous catheters (141). Coating with nanomaterials or nanoparticles has been studied as a possible improvement in the effect of reducing the occurrence of these infections. Although this technology has been developed with the main focus of eliminating bacteria, the use of silane system coatings on titanium and zirconia specimens has been described in a process of implant modification for the elimination of *Candida albicans* biofilms (142).

Given the absence of a real solution, new fields have been explored in order to find a different path to eliminate *Candida* biofilms. Research has been developed to find new natural products or synthetic peptides that might have an antifungal action. The study of different compounds has revealed interesting candidates with antifungal activity. Such is the case of phenylpropanoids and terpenoids of plant origin (143, 144), and phenazines produced by *P. aeruginosa* (145), which inhibit yeast-to-hyphal transition and biofilm formation in *C. albicans*. Besides these compounds, several high-throughput screenings have been performed trying to identify the best candidates to inhibit *Candida* biofilms. Siles et al. have screened a library of 1,200 off-patent drugs approved by the Food and Drug Administration, finding 38 active compounds against *C. albicans* biofilms. Another high-throughput screening has identified the SM21 compound as the best inhibitor of yeast-to-hypha transition, from a library of 50,240 small molecules, not having any toxic effect when tested in different human cell lines (146). Moreover, some synthetic peptides also display significant antifungal activity, as is the case of KSL-W. This peptide significantly affects the growth, yeast-to-hypha transition and biofilm formation of *C. albicans* (147). Karlsson et al. have used an antifungal β-peptide in coating nanotechnology. This approach was based on the design of polymer films that enable the localized release of the β-peptide from the surface. When testing film surfaces coated with this polymer, a significant reduction of cell viability, metabolic activity and hyphal elongation was observed (148).

Besides coating the surfaces with given molecules, another strategy is based on the modification of polymers incorporated in medical devices. Synthesis of water insoluble and organo-soluble polyethylenimine derivates with subsequent quaternization of N-methyl polyethylenimine with alkyl bromides was evaluated regarding the antifungal activity obtained. The resulting cationic polymers are capable of inhibiting *C. albicans, C. tropicalis*, and *C. dubliniensis* growth very effectively. The mode of action of these polymers is believed to be related to the disruption of membrane integrity (149). Furthermore, chitosan hydrogel is another available polymer from natural origin that may be used to modify the surface of medical devices. Chitosan is very effective in impairing biofilm formation of *C. albicans, C. parapsilosis, C. glabrata, C. tropicalis, C. krusei*, and *C. guilliermondii*, having been tested positively in a *in vivo* catheter mouse model against *C. parapsilosis* biofilm formation (150).

Although most antifungal agents fail to eliminate *Candida* biofilms, there is still hope thanks to some relatively new drug formulations. That is the case of lipid formulations of polyene antifungals, such as liposomal amphotericin B and amphotericin B lipid complex. When used against *C. albicans* biofilms formed in a bioprosthetic model, the results show MICs like those obtained with planktonic cells. Also, the echinocandins, caspofungin and micafungin, have effective action against *C. albicans* and *C. parapsilosis* biofilms in the same model (98). Interestingly, when biofilm precursor planktonic cells were exposed to subinhibitory levels of voriconazole, nystatin or chlorhexidine, and subsequently left to form biofilm, the same antifungal resistance of biofilms was not observed. In fact, a significant decrease of the capacity of the cells to form biofilm was observed (98). Another interesting drug that has been discovered to have a role in acting against *in vitro* filamentous growth in *C. albicans* and against biofilms in *C. albicans, C. glabrata*, and *C. parapsilosis* is acetylsalicylic acid, usually known as aspirin. A decrease in biofilm formation was observed for aspirin concentrations between 0.43 and 1.73 mM (151).

An alternative approach to eradicate *Candida* biofilms consists in the photodynamic inactivation. This technique is based on the use of visible light and a nontoxic dye called photosensitizer that when activated leads to the production of reactive oxygen species which subsequently kill the targeted microbial cells due to the damage provoked on DNA, proteins, and/or cell membrane. Different photosensitizers have been tested against *Candida* biofilms demonstrating the great potential of this technique as an antimicrobial therapy. The photosensitizer toluidine blue (0.1 mg/mL) has been used successfully, reducing up to 60% the formation of *C. albicans* biofilms (152). Another promising photosensitizer is methylene blue, capable of preventing *C. parapsilosis* biofilm formation *ex vivo* on mouse tongues after being activated by red LED light (153). Interestingly, the combination of photodynamic inactivation strategy, using toluidine blue, together with chitosan, results in an enhancement of the effect of both approaches against *Candida* biofilms (154). The nontoxicity of the dyes and the low cost of the technique highlight the great potential of this alternative approach (155).

## Conclusion and Future Perspectives

This review highlights the overwhelming complexity of the intracellular mechanisms leading to the formation of *Candida* biofilms, including those controlling adhesion, changes in cell morphology and EPS production, especially considering that these have been shown to be in many cases species, or even strain, specific. Additionally, external factors, including the surface where the biofilm forms, which may be a layer of epithelial cells or very diversified plastic polymers, the cocktail of nutrients and inhibitors that are present in the surrounding environment or the presence of other synergistic or antagonistic microorganisms, have a tremendous effect on the final characteristics of the formed biofilm.

All this variability poses concerning challenges from the clinical point of view, leading to the successful persistence of *Candida* infections associated with high mortality rates. All the tactics against *Candida* biofilms implemented so far are only able to address this problem partially. Therefore, treatment and prevention of *Candida* biofilms must suffer improvements so that an effective solution might be applied. As discussed, more recent strategies have the potential to be improved although continuous research for new therapies or for the best combination between the available ones are also possible approaches. Nonetheless, the specific knowledge on all the mechanisms behind biofilm formation and antifungal resistance in each *Candida* species will be crucial for the delineation of a more effective strategic plan in the fight against *Candida* infections.

Despite all gathered knowledge, there is still a lot to be scrutinized, including many aspects regarding the formation of biofilms by non-*albicans Candida* species, whose study is decades behind that of *C. albicans*. Table 1 and Figure 2 highlight a lot of the blanks that are still to be filled regarding the specific mechanisms used by *C. glabrata, C. parapsilosis*, and *C. tropicalis*, which are emerging as important human pathogens. Additionally, it will also be highly relevant to ascertain the differences between biofilms formed by commensal *Candida* populations and those related to increased pathogenesis and persistent infections.

## Author Contributions

MC gathered the bibliographic material. MC and MT wrote the manuscript. MT made the conceptual design of the manuscript and guided its production.

## Conflict of Interest Statement

The authors declare that the research was conducted in the absence of any commercial or financial relationships that could be construed as a potential conflict of interest.
